# A comparative study of methylglyoxal metabolism in trypanosomatids

**DOI:** 10.1111/j.1742-4658.2008.06788.x

**Published:** 2009-01

**Authors:** Neil Greig, Susan Wyllie, Stephen Patterson, Alan H Fairlamb

**Affiliations:** Division of Biological Chemistry and Drug Discovery, Wellcome Trust Biocentre, College of Life Sciences, University of DundeeUK

**Keywords:** glyoxalase, lactate, methylglyoxal metabolism, *Trypanosoma brucei*, trypanothione

## Abstract

The glyoxalase system, comprising the metalloenzymes glyoxalase I (GLO1) and glyoxalase II (GLO2), is an almost universal metabolic pathway involved in the detoxification of the glycolytic byproduct methylglyoxal to d-lactate. In contrast to the situation with the trypanosomatid parasites *Leishmania major* and *Trypanosoma cruzi*, this trypanothione-dependent pathway is less well understood in the African trypanosome, *Trypanosoma brucei*. Although this organism possesses a functional GLO2, no apparent GLO1 gene could be identified in the *T. brucei* genome. The absence of GLO1 in *T. brucei* was confirmed by the lack of GLO1 activity in whole cell extracts, failure to detect a GLO1-like protein on immunoblots of cell lysates, and lack of d-lactate formation from methylglyoxal as compared to *L. major* and *T. cruzi*. *T. brucei* procyclics were found to be 2.4-fold and 5.7-fold more sensitive to methylglyoxal toxicity than *T. cruzi* and *L. major*, respectively. *T. brucei* also proved to be the least adept of the ‘Tritryp’ parasites in metabolizing methylglyoxal, producing l-lactate rather than d-lactate. Restoration of a functional glyoxalase system by expression of *T. cruzi* GLO1 in *T. brucei* resulted in increased resistance to methylglyoxal and increased conversion of methylglyoxal to d-lactate, demonstrating that GLO2 is functional *in vivo*. Procyclic forms of *T. brucei* possess NADPH-dependent methylglyoxal reductase and NAD^+^-dependent l-lactaldehyde dehydrogenase activities sufficient to account for all of the methylglyoxal metabolized by these cells. We propose that the predominant mechanism for methylglyoxal detoxification in the African trypanosome is via the methylglyoxal reductase pathway to l-lactate.

The protozoan parasites *Trypanosoma cruzi*, *Trypanosoma brucei* and *Leishmania* spp. are the causative agents of the human infections Chagas’ disease, sleeping sickness and leishmaniasis, respectively. These diseases are responsible for more than 120 000 fatalities annually and the loss of over 4 600 000 disease-adjusted life-years [1]. Some of the poorest areas of the world are afflicted by these vector-borne parasites, and the accompanying economic burden is a major obstacle to improving human health [2]. Current treatments for protozoan diseases suffer from a range of problems, including severe toxic side effects [3] and acquired drug resistance [4,5]. To compound these difficulties, many of the current chemotherapeutic treatments require lengthy periods of hospitalization and are prohibitively expensive [1]. Therefore, novel drug targets and more effective drug treatments are required to combat these problems.

Metabolic pathways that are absent from, or significantly different to, host pathways are logical starting points for drug discovery [2,6]. Trypanosomatids are uniquely dependent upon trypanothione [*N*^1^*N*^8^-bis(glutathionyl)spermidine] as their principal thiol, in contrast to most other organisms (including their mammalian hosts), which utilize glutathione (γ-l-glutamyl-l-cysteinylglycine) [7]. This dithiol is primarily responsible for the maintenance of thiol-redox homeostasis within trypanosomatids, and is crucially involved in the protection of parasites from oxidative stress [8], heavy metals [9] and xenobiotics [10]. Several enzymes involved in trypanothione biosynthesis and its downstream metabolism have been genetically and chemically validated as essential for parasite survival [11]. Consequently, trypanothione-dependent enzymes have become the focus of much anti-trypanosomatid drug discovery.

The glyoxalase system, comprising the metalloenzymes glyoxalase I (GLO1, EC 4.4.1.5) and glyoxalase II (GLO2, EC 3.1.2.6), together with glutathione as cofactor, is a widely distributed pathway involved in metabolism of the toxic and mutagenic glycolytic byproduct methylglyoxal [12,13]. A unique trypanothione-dependent glyoxalase system has been identified in *Leishmania* spp. and *T. cruzi* [14–16]. In the first step, GLO1 isomerizes the spontaneous hemithioacetal adduct formed between trypanothione and methylglyoxal to *S*-d-lactoyltrypanothione [14]. In the second step, GLO2 catalyses hydrolysis of this ester, releasing d-lactate and regenerating trypanothione. The trypanothione-dependent glyoxalase system in these parasites differs significantly from that employed by their mammalian hosts, which depends entirely on glutathione as a thiol cofactor. These differences in substrate specificity may provide an opportunity for the specific chemotherapeutic targeting of these enzymes in the trypanosomatids. As inhibitors of the glyoxalase system have already been shown to possess both anticancer [17] and antimalarial [18] activities, it is possible that inhibition of the trypanothione-dependent glyoxalase pathway may prove toxic to trypanosomatids.

Although glyoxalase metabolism has been well defined in both *Leishmania major* and *T. cruzi*, this pathway is less well understood in *T. brucei*. Intriguingly, the recently completed *T. brucei* genome revealed that although this organism possesses a functional GLO2 [19], no apparent GLO1 gene or homologue could be identified [20]. This was unexpected, as the bloodstream form of *T. brucei* has an extremely high glycolytic flux and relies solely on substrate-level phosphorylation for ATP production [21]. Triose phosphates are a major source of methylglyoxal [12,13], and thus the reported antiproliferative effects of exogenous dihydroxyacetone [22] or endogenous modulation of triose phosphate isomerase in *T. brucei* [23] could be due to methylglyoxal toxicity. Should the absence of GLO1 from this pathogen be confirmed, it may have important implications for the viability of the glyoxalase system as a target for antitrypanosomatid chemotherapy. In this study, we attempted to further characterize the unusual methylglyoxal metabolism of *T. brucei* and directly compare it to that of *T. cruzi* and *L. major*.

## Results and Discussion

### Analysis of methylglyoxal-catabolizing enzymes in trypanosomatid cell extracts

Sequencing of the ‘Tritryp’ genomes has revealed several interesting distinctions between the cellular metabolism of *T. brucei*, *T. cruzi* and *L. major* [20]. In our current study, we sought to examine the apparent absence of a gene encoding a GLO1 homologue from the *T. brucei* genome, GLO1 being a ubiquitous enzyme required for the metabolism of methylglyoxal. Initially, the relative activities of enzymes involved in methylglyoxal metabolism were compared in these medically significant trypanosomatids. Whole cell extracts of *T. cruzi* epimastigotes, *L. major* promastigotes and *T. brucei* (bloodstream and procyclic forms) were prepared, and the activities of methylglyoxal-catabolizing enzymes were determined (Table 1). In keeping with previously published data [14,15], trypanothione-dependent GLO1 activity was detected in both *L. major* and *T. cruzi* extracts with specific activities of 85 and 42 nmol·min^−1^·mg^−1^, respectively. However, GLO1 activity could not be detected in extracts of *T. brucei* procyclic or bloodstream forms, with either trypanothione or glutathione hemithioacetals as substrate. In contrast, trypanothione-dependent GLO2 activity was detected in all cell lysates. With *S*-d-lactoyltrypanothione as a substrate, *L. major* extracts demonstrated GLO2 activity of 62.8 nmol·min^−1^·mg^−1^, over sixfold higher than that of *T. cruzi* extracts (8.8 nmol·min^−1^·mg^−1^). Despite the apparent lack of GLO1 activity, both *T. brucei* bloodstream form and procyclic extracts effectively metabolized *S*-d-lactoyltrypanothione, with specific activities of 18 and 23 nmol·min^−1^·mg^−1^, respectively. Trypanothione reductase activities were also assayed in each lysate to ensure adequate extraction of the parasites, and were in line with previously published data [24].

**Table 1:** Analysis of methylglyoxal-catabolizing activities in trypanosomatid lysates. All enzymatic activities were assayed as described in Experimental procedures, and corrected for nonenzymatic background rates. Specific activities represent the means ± SD of six determinations from two independent experiments.

	Specific activity (nmol·min^−1^·mg^−1^)			
Enzyme	*L. major*	*T. cruzi*	*T. brucei* procyclics	*T. brucei* bloodstream forms
GLOI	85.1 ± 3.8	42.3 ± 2.4	< 5	< 5
GLOII	62.8 ± 3.6	8.82 ± 0.29	17.9 ± 2.1	22.9 ± 3.4
Methylglyoxal reductase	5.3 ± 0.7	4.8 ± 0.42	9.4 ± 1.1	10 ± 2.3
Lactaldehyde dehydrogenase	0.51 ± 0.004	0.48 ± 0.02^a^	1.24 ± 0.11	< 0.4
Trypanothione reductase	266 ± 30	133 ± 5.6	39.6 ± 2.8	46.3 ± 3.9

a Activity measured in whole cell lysate.

### Western blot analyses of cell extracts

**Fig. 1:**
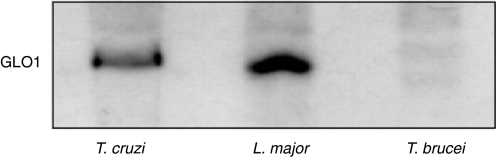
Immunoblot analysis of trypanosomatid whole cell lysates. Immunoblots of whole cell extracts (30 μg of protein in each lane) from *T. cruzi* epimastigotes, *L. major* promastigotes and *T. brucei* procyclics were probed with antiserum to *L. major* GLO1.

To confirm the absence of GLO1 from *T. brucei* at the protein level, immunoblots of trypanosomatid whole cell lysates were probed with *L. major* GLO1-specific polyclonal antiserum (Fig. 1). As expected, a protein of 16 kDa, which is equivalent to the predicted molecular mass of GLO1, reacted strongly with the antiserum in both the *L. major* and the *T. cruzi* lysates. No GLO1-like protein was detected in whole cell lysates of *T. brucei* procyclics, despite overexposure of the blot. In combination with our enzymatic analysis of cell extracts, these data confirm the absence of a functional GLO1 enzyme within *T. brucei*. This situation is not entirely without precedence. Cestode and digenean parasitic helminths have been studied that lack GLO1 while maintaining high levels of GLO2 activity [25]. One explanation for the retention of this enzyme is that *T. brucei* GLO2 has methylglyoxal-independent functions. Indeed, human GLO2 has demonstrated substrate promiscuity in efficiently hydrolysing thiol esters of simple acids such as formic acid, succinic acid and mandelic acid [13]. The identification of the true physiological substrate of *T. brucei* GLO2 will form the basis of our future studies.

### Effects of methylglyoxal on trypanosomatid growth

**Fig. 2:**
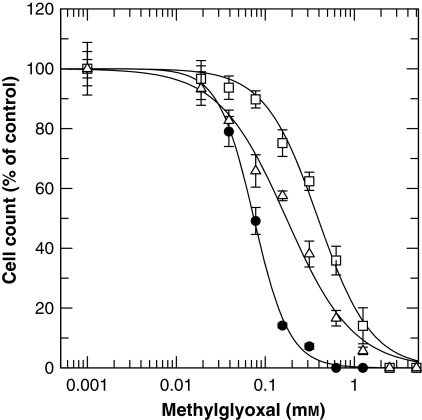
EC_50_ values for methylglyoxal against the ‘Tritryp’ trypanosomatids. The EC_50_ values for methylglyoxal against *L. major* promastigotes (open squares), *T. cruzi* epimastigotes (open triangles) and *T. brucei* procyclics (closed circles) were determined. The curves are the nonlinear fits of data using a two-parameter EC_50_ 0–100% equation provided by grafit (see Experimental procedures). EC_50_ values of 70 ± 2 methylglyoxal, 171 ± 11 and 397 ± 27 μm were determined for *T. brucei*, *T. cruzi* and *L. major* with corresponding slope factors (*m*) of 3.0, 1.6 and 1.59, respectively. Data are the means of triplicate measurements.

The absence of GLO1 from *T. brucei* suggested that these parasites may be particularly susceptible to the toxic effects of methylglyoxal. With this in mind, *T. cruzi*, *L. major* and *T. brucei* were grown in the presence of increasing methylglyoxal concentrations, and the relative growth of each culture was determined after 72 h (Fig. 2). To allow the direct comparison of the methylglyoxal sensitivity of these parasites, each cell line was adapted for growth in SDM-79 medium prior to analysis. *T. brucei* procyclics were the most sensitive to methylglyoxal toxicity, with an EC_50_ of 70 ± 2 μm, whereas *T. cruzi* epimastigotes and *L. major* promastigotes were 2.4-fold and 5.7-fold less sensitive, with EC_50_ values of 171 ± 11 and 397 ± 27 μm, respectively.

Bloodstream trypanosomes could not be adapted for growth in SDM-79 medium, and attempts to determine the methylglyoxal sensitivity of these cells in HMI-9 medium proved unsuccessful, due to the propensity of methylglyoxal to react with thiols in this culture medium. In a previous study on the curative effect of methylglyoxal in cancer-bearing mice [26], Ghosh *et al.* established the pharmacokinetic properties of methylglyoxal in blood following oral dosing. Using this methodology, we examined the effects of methylglyoxal on an *in vivo T. brucei* infection. The maximum achievable methylglyoxal concentration in blood following oral dosing of mice was 20 μm, and at this level there was no discernible effect on the progression of the parasite infection (data not shown). These results suggest that the methylglyoxal EC_50_ for bloodstream *T. brucei in vivo* is in excess of 20 μm.

### Trypanosomatid metabolism of methylglyoxal

**Fig. 3:**
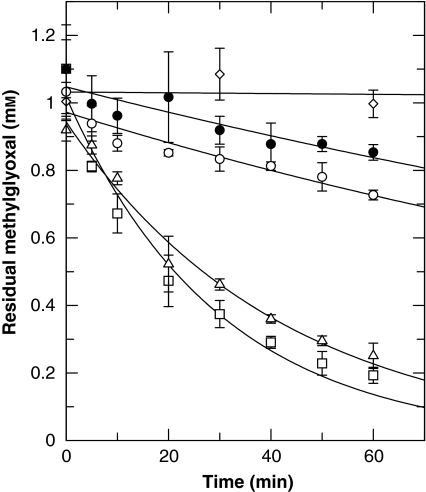
Metabolism of methylglyoxal in the ‘Tritryp’ trypanosomatids. The metabolism of methylglyoxal (1.5 mm) by mid-log *L. major* promastigotes (open squares), *T. cruzi* epimastigotes (open triangles), *T. brucei* procyclics (closed circles) and *T. brucei* bloodstream forms (open circles) was monitored over 1 h. Methylglyoxal metabolism in assay buffer in the absence of cells was also measured (open diamonds). Data are fitted to single exponential fits using equations in grafit, and are the means of triplicate measurements.

The rate of exogenous methylglyoxal metabolism by *T. cruzi*, *L. major* and *T. brucei* (bloodstream and procyclic forms) was determined (Fig. 3). Each cell line was resuspended in a minimal medium that had been preincubated with 1.5 mm methylglyoxal for 90 min. At defined intervals, culture supernatants were removed and analysed for residual methylglyoxal. In keeping with both our enzymatic analysis of whole cell lysates and EC_50_ data, *L. major* promastigotes dealt with exogenous methylglyoxal most efficiently, with an initial rate of 67 nmol·min^−1^·mL^−1^. In comparison, *T. cruzi* epimastigotes were considerably less effective at metabolizing methylglyoxal (47.6 nmol·min^−1^·mL^−1^). However, *T. brucei* procyclics and bloodstream forms proved to be the least adept at dealing with this toxic oxoaldehyde, metabolizing methylglyoxal with initial rates of 7.4 nmol·min^−1^·mL^−1^ and 9.8 nmol·min^−1^·mL^−1^, respectively. These results suggest that although *T. brucei* is predicted to be the most vulnerable of the ‘Tritryp’ trypanosomatids to methylglyoxal toxicity, it can effectively metabolize methylglyoxal despite the absence of a complete glyoxalase pathway.

### Products of trypanosomatid metabolism of methylglyoxal

In all studies to date, the principal product of thiol-dependent metabolism of methylglyoxal has been d-lactate [27–29]. Consequently, methylglyoxal-treated parasites were monitored for the production of lactate, using d-lactate and l-lactate dehydrogenase-based assays (Table 2). As expected, both *L. major* and *T. cruzi* cells produced considerable amounts of d-lactate following exposure to methylglyoxal, accounting for approximately 30% of free methylglyoxal in the medium. In contrast, *T. brucei* (procyclics and bloodstream forms) produced only trace amounts of d-lactate. Instead, methylglyoxal-treated *T. brucei* procyclics and bloodstream forms produced significant quantities of the stereoisomer l-lactate (120 and 221 μm in 2 h, respectively). The sixfold higher rate of l-lactate production by bloodstream parasites in the absence of exogenous methylglyoxal reflects the extremely high glycolytic rate in this developmental form of the African trypanosome [30]. The addition of methylglyoxal marginally decreased the amount of l-lactate detected in the supernatants of both *L. major* and *T*. *cruzi* cultures. These data suggest that *T. brucei* may metabolize methylglyoxal by an alternative pathway.

**Table 2:** Comparison of methylglyoxal-stimulated d-lactate and l-lactate production by trypanosomatids. Parasites were incubated with or without methylglyoxal for 2 h prior to analysis. Data represent the mean ± SD of triplicate determinations. See Experimental procedures for further details.

	Lactate (μm)					
	d-Lactate			l-Lactate		
Organism	Plus methylglyoxal	Minus methylglyoxal	Net	Plus methylglyoxal	Minus methylglyoxal	Net
*L. major*	385 ± 9	49 ± 9	337	7 ± 0.6	12 ± 0.1	−5
*T. cruzi*	303 ± 3	8 ± 0.8	295	9 ± 0.3	13 ± 0.1	−4
*T. brucei* procyclics	18 ± 0.2	8 ± 0.1	11	141 ± 5	21 ± 0.1	120
*T. brucei* bloodstream forms	68 ± 9	50 ± 2	18	355 ± 42	134 ± 18	221

In a previous study [31], Ghoshal *et al.* identified NADPH-dependent methylglyoxal reductase activity in *Leishmania donovani* promastigotes. These parasites were shown to metabolize approximately 1.2% of the exogenous methylglyoxal added to cultures via this reductase, generating l-lactaldehyde as an end-product. In view of the generation of considerably higher levels of l-lactate by methylglyoxal-treated *T. brucei*, we hypothesized that methylglyoxal reductase activity may be elevated in *T. brucei* to compensate for the absence of GLO1. Indeed, when NADPH-dependent methylglyoxal reductase activity was measured in all three trypanosomatid cell lysates, a twofold higher reductase activity was observed in *T. brucei* procyclic and bloodstream extracts, respectively, than that seen in *L. major* and *T. cruzi* cells (Table 1). If we consider that procyclics metabolize exogenous methylglyoxal at a rate of 7.4 nmol·min^−1^ per 10^8^ cells (Fig. 3), and assuming that 10^8^ cells is equivalent to 1 mg of protein [32], this elevated methylglyoxal reductase activity could conceivably account for all methylglyoxal metabolism in *T. brucei*. Although a *T. brucei* methylglyoxal reductase has yet to be identified, two putative aldo-keto reductase genes (Tb927.2.5180 and Tb11.02.3040), whose protein products are members of the same aldo-keto reductase superfamily as methylglyoxal reductase, have been annotated in the genome. To date, attempts to express these genes as soluble recombinant proteins have proved unsuccessful. In mammalian cells, methylglyoxal can also be detoxified by two methylglyoxal dehydrogenase enzymes (oxoaldehyde dehydrogenase and betaine aldehyde dehydrogenase) [33]. No homologues of these enzymes were identified in the *T. brucei* genome, and neither NAD^+^-dependent nor NADP^+^-dependent methylglyoxal dehydrogenase activities were detected in *T. brucei* extracts (data not shown).

To complete the metabolism of l-lactaldehyde to lactate, *T. brucei* would require a functional l-lactaldehyde dehydrogenase. Although lactaldehyde dehydrogenase activity has previously been detected in *L. donovani* cell lysates [31], it has yet to be identified in either *T. cruzi* or in *T. brucei*. Using l-lactaldehyde as a substrate, l-lactaldehyde dehydrogenase activity was measured in all three insect-stage trypanosomatid cell lysates (Table 1), and was found to be relatively similar in *L. major* and *T. cruzi* lysates (0.51 ± 0.004 and 0.48 ± 0.02 nmol·min^−1^·mg^−1^, respectively). In comparison, l-lactaldehyde dehydrogenase activity was found to be elevated approximately 2.4-fold in *T. brucei* procyclic cell lysates (1.24 ± 0.11 nmol·min^−1^·mg^−1^). However, activity could not be detected in the bloodstream stage of the parasite. These studies confirm that procyclic *T. brucei* organisms are capable of metabolizing methylglyoxal, via a methylglyoxal reductase-dependent pathway, to l-lactate; however, it remains to be seen whether this is the predominant pathway for methylglyoxal detoxification in these cells. Our failure to detect NAD^+^-dependent l-lactaldehyde dehydrogenase activity in *T. brucei* bloodstream forms may be due to technical reasons, such as NADH oxidation via the glycerophosphate oxidase system masking the formation of NADH.

### Expression of *T. cruzi* GLO1 (*Tc*GLO1) in *T. brucei*

**Fig. 4:**
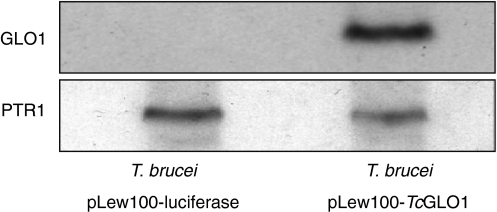
*Tc*GLO1 expression in *T. brucei* procyclics. Immunoblots of cell extracts of *T. brucei* procyclics transfected with either pLew100–luciferase or pLew100–*Tc*GLO1 were probed with antiserum to *L. major* GLO1 and *T. brucei* pteridine reductase 1 (PTR1) (1 × 10^7^ parasites in each lane). Cells were induced with tetracycline for 24 h prior to analysis.

Can *T. brucei* utilize a complete glyoxalase system? To address this question, a tetracycline-inducible pLew100–*Tc*GLO1 construct was generated and transfected into both bloodstream and procyclic cells. Western blot analysis of transgenic parasites, following induction with tetracycline, confirmed the expression of a 16-kDa protein that reacted strongly with GLO1-specific antiserum (Fig. 4; bloodstream data not shown). This protein was not evident in cells transfected with an unrelated vector (pLew100–luciferase). Antiserum against *T. brucei* pteridine reductase 1 was used to establish equal loading of samples. The expression of recombinant *T*cGLO1 in procyclics and bloodstream forms was confirmed when GLO1 activity (23.0 ± 1.9 and 38.2 ± 1.9 nmol·min^−1^·mg^−1^, respectively) was detected in cell extracts. Indeed, the rate of exogenous methylglyoxal metabolism in these transgenic *T. brucei* cell lines increased markedly, with GLO1-expressing procyclic and bloodstream cells metabolizing the toxic oxoaldehyde 1.7-fold and 2.7-fold more effectively, respectively (Table 3). Most importantly, *Tc*GLO1-expressing *T. brucei* procyclics were almost 3.5-fold less sensitive to methylglyoxal than wild-type or luciferase-expressing cells.

**Table 3:** Comparison of GLO1 activity, methylglyoxal sensitivity and methylglyoxal metabolism in *T. brucei* wild-type and transgenic cell lines. ND, not determined.

Cell line	GLO1 activity (nmol·min^−1^· mg^−1^)	EC_50_^a^ (μm)	Methylglyoxal metabolized^b^ (nmol·mL^−1^·h^−1^)
*T. brucei*			
Procyclics	< 5	53.4 ± 2.9	246 ± 21
Bloodstream forms	< 5	ND	300 ± 32
pLew100–luciferase^c^			
Procyclics	< 5	47.8 ± 3.8	197 ± 16
Bloodstream forms	< 5	ND	260 ± 28
pLew100–*Tc*GLO1^c^			
Procyclics	23.0 ± 1.9	175 ± 5.6^d^	387 ± 27
Bloodstream forms	38.2 ± 1.9	ND	810 ± 40

a Values are the weighted means of three independent experiments.

b All data represent the mean ± SD of six determinations from two independent experiments.

c Cell lines were grown in the presence of tetracycline for 24 h prior to analysis.

d *P*< 0.001 as compared to *T. brucei*.

To confirm that enhanced methylglyoxal tolerance in GLO1-expressing *T. brucei* was due to complementation of the glyoxalase system, lactate production in the supernatants of methylglyoxal-treated wild-type and transgenic cells was measured (Table 4). Whereas l-lactate levels in the supernatants of GLO1-expressing *T. brucei* (bloodstream forms and procyclics) were very similar to those of the wild-type, d-lactate production was found to be significantly higher (∼ 3-fold, *P*< 0.0001). d-Lactate levels failed to reach those seen in the supernatants of methylglyoxal-treated *L. major* and *T. cruzi*, but were sufficient to suggest that GLO1 expression in *T. brucei* procyclic and bloodstream parasites results in a complete glyoxalase system.

**Table 4:** Comparison of methylglyoxal-stimulated d-lactate and l-lactate production by wild-type and transgenic *T. brucei* cell lines. Data represents the mean ± SD of six determinations from two independent experiments.

	Lactate (μm)					
	d-Lactate			l-lactate		
Cell line	Plus methylglyoxal	Minus methylglyoxal	Net	Plus methylglyoxal	Minus methylglyoxal	Net
*T. brucei*						
Procyclics	22 ± 3	10 ± 2	12	148 ± 7	24 ± 3	124
Bloodstream forms	68 ± 9	50 ± 2	18	355 ± 42	134 ± 18	221
pLew100–luciferase						
Procyclics	17 ± 2	9 ± 1	8	134 ± 12	19 ± 2	115
Bloodstream forms	59 ± 2	48 ± 3	11	329 ± 26	118 ± 21	211
pLew100–*Tc*GLO1						
Procyclics	57 ± 4^a^	19 ± 2	38	122 ± 9	22 ± 2	100
Bloodstream forms	183 ± 24^a^	71 ± 5	112	308 ± 14	105 ± 8	203

a *P*< 0.001 as compared to *T. brucei*.

### Implications for parasite chemotherapy

**Fig. 5:**
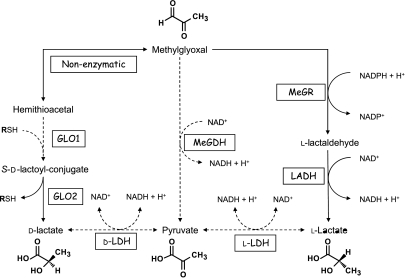
Metabolism of methylglyoxal. In *T. cruzi* and *L. major*, the principal end-product of methylglyoxal metabolism is d-lactate. In the absence of GLO1, *T. brucei* does not maintain an intact glyoxalase system, and may metabolize methylglyoxal via methylglyoxal reductase (MeGR) and lactaldehyde dehydrogenase (LADH) to l-lactate. Solid lines: confirmed metabolism in *T. brucei*. Dotted lines: metabolism absent in *T. brucei*. MeGDH, methylglyoxal dehydrogenase; LDH, lactate dehydrogenase.

Mammalian cells maintain a repertoire of four pathways for metabolism of methylglyoxal [33], whereas our studies suggest that the African trypanosome may be solely dependent upon methylglyoxal reductase (Fig. 5). The absence of a functioning glyoxalase system within *T. brucei*, recognized as the principal route of oxoaldehyde detoxification in almost all cells, is especially perplexing. As methylglyoxal is generated primarily as a byproduct of glycolysis, and African trypanosomes are entirely dependent upon glycolysis for energy, it would be reasonable to assume that *T. brucei* would preserve robust methylglyoxal-metabolizing systems. Without an intact glyoxalase pathway, these cells should be particularly vulnerable to methylglyoxal toxicity, and our current studies appear to confirm this. These findings have broad implications for the targeting of methylglyoxal metabolism for antitrypanosomatid chemotherapy. Previous studies have suggested that the contrasting substrate specificities of the human and trypanosomatid glyoxalase enzymes (GLO1 and GLO2) make them attractive targets for rational drug design [14,15,19]. Whereas this may still be the case in *T. cruzi* and *Leishmania* spp., methylglyoxal reductase is clearly a more promising drug target in the African trypanosome. Identification of the genes that encode this enzyme in *T. brucei* should now be a priority.

## Experimental procedures

### Cell lines and culture conditions

*L. major* promastigotes (Friedlin strain; WHO designation MHOM/JL/81/Friedlin), procyclic trypomastigotes of *T. brucei brucei* S427 29-13 and epimastigotes of *T. cruzi* CL Brener (genome project standard clone) were adapted for growth in SDM-79 medium supplemented with 10% fetal bovine serum (Gibco, Paisley, UK) and haemin (100 mg·L^−1^). *L. major* promastigotes were grown at 24 °C with shaking, and *T. brucei* and *T. cruzi* were cultured at 28 °C. *T. brucei* bloodstream forms were cultured at 37 °C in modified HMI9 medium (56 μm 1-thioglycerol was substituted for 200 μm 2-mercaptoethanol) supplemented with 2.5 μg·mL^−1^ G418 to maintain expression of T7 RNA polymerase and the tetracycline repressor protein [34].

In order to directly compare the effects of methylglyoxal on the growth of these trypanosomatids, triplicate cultures containing methylglyoxal were seeded at 5 × 10^5^ parasites per mL. As methylglyoxal interferes with the Alamar blue assay for viable cells, cell densities were determined using the CASY Model TT cell counter (Schärfe, Renlingen, Germany) after culture for 72 h. Concentrations of inhibitor causing a 50% reduction in growth (EC_50_) were determined using the following two-parameter equation by nonlinear regression using grafit: 
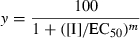
 where the experimental data were corrected for background cell density and expressed as percentages of the uninhibited control cell density. In this equation, [I] represents inhibitor concentration, and *m* is the slope factor.

### Analysis of methylglyoxal-catabolizing enzymes in trypanosomatid cell lysates

*L. major* promastigote*s* (2 × 10^7^ mL^−1^, 1 L), *T. cruzi* epimastigotes (3 × 10^7^ mL^−1^, 1 L) and *T. brucei brucei* procyclics (2 × 10^7^ mL^−1^, 1 L) were pelleted by centrifugation (1600 ***g***, 10 min, 4 °C), washed twice in 20 mm Tris (pH 7.0) containing 0.1 mm sucrose, and resuspended in cell lysis buffer (10 mm potassium phosphate, pH 7.0). For biological safety, parasites were inactivated by three cycles of freezing and thawing, before lysis under pressure (30 kpsi) using a one-shot cell disruptor (Constant Systems, Daventry, UK). *T. brucei* bloodstream forms (4 × 10^9^ cells), harvested from rats as previously described [35], were lysed using an alternative method. Cells were pelleted by centrifugation (800 ***g***, 10 min, 4 °C), washed once in PSG buffer [NaCl/P_i_, pH 8.0, 1.5% (w/v) glucose and 0.5 mg·mL^−1^ BSA], resuspended in ice-cold de-ionized dH_2_O, (500 μL) and vortexed. Lysed bloodstream trypanosomes were then incubated on ice for 10 min prior to the addition of 2× lysis buffer (500 μL) and further vortexing. From this point, all lysates were treated in an identical manner. Following centrifugation (800 ***g***, 20 min, 4 °C), cell supernatants were collected and dialysed against 50 mm Hepes (pH 7.0) with 25 mm NaCl and 150 μm 2-mercaptoethanol at 4 °C to remove components of less than 3.5 kDa. The protein concentration of each lysate was determined using Bradford reagent (Bio-Rad, Hemel Hempstead, UK). GLO1 activity in the trypanosomatid cell lysates was measured by monitoring the formation of *S*-d-lactoyltrypanothione spectrophotometrically at 240 nm [14]. Trypanothione and methylglyoxal were preincubated at 25 °C for 10 min in 50 mm Hepes (pH 7.0) plus 25 mm NaCl, 50 μm adduct, and 100 μm free thiol. Reactions were initiated with enzyme extract. Methylglyoxal reductase and GLO2 activities were determined as previously described [14,31,36]. The activity of trypanothione reductase, used as a control enzyme, was assayed as previously described [37].

### l-Lactaldehyde dehydrogenase activity in trypanosomatids

l-Lactaldehyde, the substrate of l-lactaldehyde dehydrogenase, was prepared from d-threonine, as previously described [38]. Briefly, 25 mmol of d-threonine, 9.1 g of ninhydrin and 600 mL of 0.05 m sodium citrate buffer (pH 5.4) were combined and boiled for 15 min with continual stirring. After being cooled to room temperature, the mixture was filtered and treated with sufficient Dowex 1-X8 resin (bicarbonate form) to raise the pH to 6.5. After stirring for a further 2–3 h, the resin was again filtered, and the filtrate was adjusted to pH 4.0 by the addition of Dowex 50 resin (hydrogen ion form). Following filtration, the filtrate was concentrated down to 50–100 mL using a rotary evaporator. The resulting concentrate was then sequentially treated with Dowex 1-X8 and Dowex 50 resins, as previously described, and further concentrated to 20–30 mL. Dowex 1-X8 resin was then added to the concentrated filtrate in batches until the solution was colourless, and the pH was adjusted to 7.5. The l-lactaldehyde yield from this reaction was determined by monitoring NADH production at 340 nm following incubation with aldehyde dehydrogenase from baker’s yeast (Fluka, Gillingham, UK). Reactions were performed in 100 mm Tris/HCl (pH 8.5), 3 mm NAD^+^ and 10 units of aldehyde dehydrogenase. The purity of the synthetic l-lactaldehyde was analysed by liquid chromatography–MS. Samples were derivatized with excess 2,4-dinitrophenylhydrazine (Fluka) in 5 mm HCl, diluted with acetonitrile/water (1 : 1), and analysed by liquid chromatography–MS (Phenomenex Gemini C18 column, 50 × 3.0 mm, 5 μm particle size; mobile phase, water/acetonitrile + 0.1% HCOOH 80 : 20 to 5 : 95 over 3.5 min, and then held for 1.5 min; flow rate 0.5 mL·min^−1^). LC-MS analysis detected the expected lactaldehyde hydrazone plus an additional hydrazone (the contaminant was not present in the underivatized l-lactaldehyde preparation, or the 2,4-dinitrophenylhydrazine reagent). The mass and retention time of the contaminating hydrazone was consistent with the impurity in the l-lactaldehyde preparation being acetone or propionaldehyde (as shown by comparison with the hydrazones of acetone and propionaldehyde synthesized as described above). Biochemical assays on *L. major* cell-free extracts indicated that neither acetone nor propionaldehyde was responsible for the observed activity.

l-Lactaldehyde dehydrogenase activity was measured in soluble trypanosomatid extracts, prepared as above, except that a further centrifugation (100 000 ***g***, 1 h, 4 °C) and dialysis step was introduced prior to analysis. Activity was measured at 27 °C in 100 mm Tris/HCl (pH 8.5) with 3 mm NAD^+^ and 1 mm l-lactaldehyde [39]. Trypanosomatid extracts were incubated with NAD^+^ at 27 °C for 5 min, prior to the initiation of the reaction with l-lactaldehyde. Reactions were monitored at 340 nm for the formation of NADH.

### Western blot analyses of trypanosomatid cell extracts

Polyclonal antisera against *L. major* GLO1 were raised in adult male Wistar rats. An initial injection of 100 μg of purified antigen, emulsified in complete Freund’s adjuvant, was followed by two identical booster injections of antigen emulsified in Freund’s incomplete adjuvant at 2 week intervals.

Trypanosomatid whole cell extracts (30 μg) were separated by SDS/PAGE and subsequently transferred onto nitrocellulose. After blocking with 7% skimmed milk in NaCl/P_i_ for 1 h, blots were incubated with *L. major* GLO1 polyclonal antiserum (1 : 700 dilution) for 1 h, washed in NaCl/P_i_ containing 0.1% (v/v) Tween-20, and then incubated with a secondary antibody [rabbit anti-(rat IgG)] (Dako, Ely, UK; 1 : 10 000 dilution). Immunoblots were developed using the ECL plus (enhanced chemiluminescence) system from Amersham Biosciences (Piscataway, NJ, USA).

### Analysis of methylglyoxal metabolism in trypanosomatids

Mid-log *L. major* promastigote*s*, *T. cruzi* epimastigotes and *T. brucei* procyclics (4 × 10^8^ cells) were pelleted by centrifugation (1600 ***g***, 10 min, 4 °C) and washed in a maintenance medium (250 mm sucrose, 25 mm Tris, pH 7.4, 1 mm EDTA, 8 g·L^−1^ glucose, and 0.5 mg·mL^−1^ BSA). Cells were then resuspended at 1 × 10^8^ mL^−1^ in maintenance medium that had been preincubated with 1.5 mm methylglyoxal for 90 min prior to resuspension. In the case of *T. brucei* bloodstream forms, cells were pelleted by centrifugation (800 ***g***, 10 min, 4 °C), and washed in PSG buffer with 0.5 mg·mL^−1^ BSA. Bloodstream trypanosomes were then resuspended at 1 × 10^8^ mL^−1^ in PSG buffer with 0.5 mg·mL^−1^ BSA that had been preincubated with 1.5 mm methylglyoxal for 90 min prior to resuspension. In all cases, cell viability was monitored by visibly checking motility throughout the experiment. Metabolism of methylglyoxal by these cells was determined by measuring the methylglyoxal concentration in cell-free assay buffer. At defined intervals, aliquots were removed, cells were pelleted at 16 000 ***g*** for 5 min, and the supernatants were analysed for residual methylglyoxal by the semicarbizide assay [14].

The production of lactate by methylglyoxal-treated mid-log *L. major* promastigotes, *T. cruzi* epimastigotes and both *T. brucei* procyclic and bloodstream trypanosomes (2 × 10^8^ cells) was determined. Cells were incubated with 1.5 mm methylglyoxal in an identical manner to that previously described for the methylglyoxal metabolism studies. Following a 2 h incubation, cells were pelleted (16 000 ***g***, 5 min), and supernatants were assayed without further treatment by the addition of either d-lactaldehyde dehydrogenase or l-lactaldehyde dehydrogenase, as per the manufacturer’s instructions. The amount of NADH formed was measured at 340 nm, and the limit of detection for these assays was determined to be 1 μm.

### Cloning and expression of recombinant *Tc*GLO1 in *T. brucei*

The *T. cruzi* GLO1 gene (Tc00.1047053510659.240) was amplified by PCR from genomic DNA using the sense primer 5′-*AAGCTT*ATGTCAACACGACGACTTATGCACA-3′ and the antisense primer 5′-*GGATCC*GGATCCTTAAGCCGTTCCCTGTTC-3′ with additional *Hin*dIII and *Bam*HI restriction sites (italicised), respectively. The PCR product was then cloned into pCR-Blunt II-TOPO (Invitrogen) and sequenced. The pCR-Blunt II-TOPO–*Tc*GLO1 construct was then digested with *Hin*dIII and *Bam*HI, and the fragment was ligated into the tetracycline-inducible expression vector pLew100 [40], resulting in a pLew100–*Tc*GLO1 construct.

*T. brucei brucei* procyclics [40], modified to express both T7 polymerase and the tetracycline repressor protein, were transfected with either pLew100–*Tc*GLO1 or the control vector pLew100–luciferase, as previously described [41]. Following transfection, cells were grown in SDM-79 medium in the presence of 50 μg·mL^−1^ hygromycin, 15 μg·mL^−1^ gentamicin and 2.5 μg·mL^−1^ phleomycin. *T. brucei* bloodstream forms were also transfected with the pLew100–*Tc*GLO1 vector or the pLew100–luciferase vector, as previously described [41,42], and subsequently cultured in HMI9 medium supplemented with 2.5 μg·mL^−1^ G418, to maintain expression of T7 RNA polymerase and the tetracycline repressor protein, and 2.5 μg·mL^−1^ phleomycin. Methylglyoxal metabolism in the transfected cell lines was analysed, as previously described, following induction of recombinant protein expression with tetracycline (2 μg·mL^−1^, 24 h).

## Abbreviations

GLO1: glyoxalase I
GLO2: glyoxalase II
*Tc*GLO1: *Trypanosoma cruzi* glyoxalase I

## References

[1] Stuart KD, Brun R, Croft SL, Fairlamb AH, Gurtler RE, McKerrow JH, Reed S, Tarleton RL (2008). Kinetoplastids: related protozoan pathogens, different diseases. J Clin Invest.

[2] Fairlamb AH (2002). Metabolic pathway analysis in trypanosomes and malaria parasites. Philos Trans R Soc Lond B Biol Sci.

[3] Wyllie S, Fairlamb AH (2006). Differential toxicity of antimonial compounds and their effects on glutathione homeostasis in a human leukaemia monocyte cell line. Biochem Pharmacol.

[4] Croft SL, Sundar S, Fairlamb AH (2006). Drug resistance in Leishmaniasis. Clin Microbiol Rev.

[5] Barrett MP, Fairlamb AH (1999). The biochemical basis of arsenical–diamidine cross-resistance in African trypanosomes. Parasitol Today.

[6] Frearson JA, Wyatt PA, Gilbert IH, Fairlamb AH (2007). Target assessment for antiparasitic drug discovery. Trends Parasitol.

[7] Fairlamb AH, Blackburn P, Ulrich P, Chait BT, Cerami A (1985). Trypanothione: a novel bis(glutathionyl)spermidine cofactor for glutathione reductase in trypanosomatids. Science.

[8] Ariyanayagam MR, Fairlamb AH (2001). Ovothiol and trypanothione as antioxidants in trypanosomatids. Mol Biochem Parasitol.

[9] Mandal G, Wyllie S, Singh N, Sundar S, Fairlamb AH, Chatterjee M (2007). Increased levels of thiols protect antimony unresponsive *Leishmania donovani* field isolates against reactive oxygen species generated by trivalent antimony. Parasitology.

[10] Fairlamb AH, Cerami A (1992). Metabolism and functions of trypanothione in the Kinetoplastida. Annu Rev Microbiol.

[11] Krauth-Siegel RL, Comini MA (2008). Redox control in trypanosomatids, parasitic protozoa with trypanothione-based thiol metabolism. Biochim Biophys Acta.

[12] Thornalley PJ (1993). The glyoxalase system in health and disease. Mol Aspects Med.

[13] Vander Jagt DL, Dolphin D, Poulson R, Avramovic O (1989). The glyoxalase system. Glutathione: Chemical, Biochemical and Medical Aspects. Part A.

[14] Vickers TJ, Greig N, Fairlamb AH (2004). A trypanothione-dependent glyoxalase I with a prokaryotic ancestry in *Leishmania major*. Proc Natl Acad Sci USA.

[15] Greig N, Wyllie S, Vickers TJ, Fairlamb AH (2006). Trypanothione-dependent glyoxalase I in *Trypanosoma cruzi*. Biochem J.

[16] Padmanabhan PK, Mukherjee A, Madhubala R (2006). Characterization of the gene encoding glyoxalase II from *Leishmania donovani*: a potential target for anti-parasite drug. Biochem J.

[17] Thornalley PJ, Ladan MJ, Ridgway SJS, Kang Y (1996). Antitumor activity of S-(p-bromobenzyl)glutathione diesters *in vitro*: a structure–activity study. J Med Chem.

[18] Thornalley PJ, Strath M, Wilson RJM (1994). Antimalarial activity *in vitro* of the glyoxalase I inhibitor diester, *S*-p-bromobenzylglutathione diethyl ester. Biochem Pharmacol.

[19] Irsch T, Krauth-Siegel RL (2004). Glyoxalase II of African trypanosomes is trypanothione-dependent. J Biol Chem.

[20] Berriman M, Ghedin E, Hertz-Fowler C, Blandin G, Renauld H, Bartholomeu DC, Lennard NJ, Caler E, Hamlin NE, Haas B (2005). The genome of the African trypanosome *Trypanosoma brucei*. Science.

[21] Fairlamb AH, Opperdoes FR, Morgan MJ (1986). Carbohydrate metabolism in African trypanosomes, with special reference to the glycosome. Carbohydrate Metabolism in Cultured Cells.

[22] Uzcategui NL, Carmona-Gutierrez D, Denninger V, Schoenfeid C, Lang F, Figarella K, Duszenko M (2007). Antiproliferative effect of dihydroxyacetone on *Trypanosoma brucei* bloodstream forms: cell cycle progression, subcellular alterations, and cell death. Antimicrob Agents Chemother.

[23] Helfert S, Estevez AM, Bakker B, Michels P, Clayton C (2001). Roles of triosephosphate isomerase and aerobic metabolism in *Trypanosoma brucei*. Biochem J.

[24] Vickers TJ, Fairlamb AH (2004). Trypanothione *S*-transferase activity in a trypanosomatid ribosomal elongation factor 1B. J Biol Chem.

[25] Brophy PM, Barrett J (1990). Glutathione transferase in helminths. Parasitology.

[26] Ghosh M, Talukdar D, Ghosh S, Bhattacharyya N, Ray M, Ray S (2006). *In vivo* assessment of toxicity and pharmacokinetics of methylglyoxal – augmentation of the curative effect of methylglyoxal on cancer-bearing mice by ascorbic acid and creatine. Toxicol Appl Pharmacol.

[27] Martins AM, Cordeiro CA, Ponces Freire AM (2001). In situ analysis of methylglyoxal metabolism in *Saccharomyces cerevisiae*. FEBS Lett.

[28] Shih MJ, Edinger JW, Creighton DJ (1997). Diffusion-dependent kinetic properties of glyoxalase I and estimates of the steady-state concentrations of glyoxalase-pathway intermediates in glycolyzing erythrocytes. Eur J Biochem.

[29] Atlante A, de Bari L, Valenti D, Pizzuto R, Paventi G, Passarella S (2005). Transport and metabolism of D-lactate in Jerusalem artichoke mitochondria. Biochim Biophys Acta Bioenerg.

[30] Ter Kuile BH, Opperdoes FR (1991). Glucose uptake by *Trypanosoma brucei*. Rate-limiting steps in glycolysis and regulation of the glycolytic flux. J Biol Chem.

[31] Ghoshal K, Banerjee AB, Ray S (1989). Methylglyoxal-catabolizing enzymes of *Leishmania donovani* promastigotes. Mol Biochem Parasitol.

[32] Le Quesne SA, Fairlamb AH (1996). Regulation of a high-affinity diamine transport system in *Trypanosoma cruzi* epimastigotes. Biochem J.

[33] Vander Jagt DL, Hunsaker LA (2003). Methylglyoxal metabolism and diabetic complications: roles of aldose reductase, glyoxalase-1, betaine aldehyde dehydrogenase and 2-oxoaldehyde dehydrogenase. Chem Biol Interact.

[34] Hirumi H, Hirumi K (1989). Continuous cultivation of *Trypanosoma brucei* blood stream forms in a medium containing a low concentration of serum protein without feeder cell layers. J Parasitol.

[35] Lanham SM (1968). Separation of trypanosomes from the blood of infected rats and mice by anion-exchangers. Nature.

[36] Martins AM, Cordeiro C, Freire AP (1999). Glyoxalase II in *Saccharomyces cerevisiae*: *in situ* kinetics using the 5,5′-dithiobis(2-nitrobenzoic acid) assay. Arch Biochem Biophys.

[37] Cunningham ML, Fairlamb AH (1995). Trypanothione reductase from *Leishmania donovani*– purification, characterisation and inhibition by trivalent antimonials. Eur J Biochem.

[38] Zagalak B, Frey PA, Karabats GL, Abeles RH (1966). Stereochemistry of conversion of D and L 1,2-propanediols to propionaldehyde. J Biol Chem.

[39] Ray S, Ray M (1984). Oxidation of lactaldehyde by cytosolic aldehyde dehydrogenase and inhibition of cytosolic and mitochondrial aldehyde dehydrogenase by metabolites. Biochim Biophys Acta.

[40] Wirtz E, Leal S, Ochatt C, Cross GAM (1999). A tightly regulated inducible expression system for conditional gene knock-outs and dominant-negative genetics in *Trypanosoma brucei*. Mol Biochem Parasitol.

[41] Burkard G, Fragoso CM, Roditi I (2007). Highly efficient stable transformation of bloodstream forms of *Trypanosoma brucei*. Mol Biochem Parasitol.

[42] Sienkiewicz N, Jaroslawski S, Wyllie S, Fairlamb AH (2008). Chemical and genetic validation of dihydrofolate reductase-thymidylate synthase as a drug target in African trypanosomes. Mol Microbiol.

